# Do Changes in Language Context Affect Visual Memory in Bilinguals?

**DOI:** 10.3389/fnhum.2019.00364

**Published:** 2019-10-17

**Authors:** Scott R. Schroeder

**Affiliations:** Department of Speech-Language-Hearing Sciences, Hofstra University, Hempstead, NY, United States

**Keywords:** language, memory, bilingualism, context, multisensory

## Abstract

Language is often present when people are encoding visual memories. For bilinguals, this language context can have different forms (i.e., Language A, Language B, or both Language A and B), and can change over the course of events. The current study examined whether a change in language context during a visual event or between visual events affects a bilingual’s ability to remember visual information. English-Spanish bilinguals and control participants encoded three lists of novel shapes amid different task-irrelevant language contexts. Following each list, participants completed a free recall test in which they drew the novel shapes they remembered. Results indicated that a change in language context between events, but not during events, affected visual memory. Specifically, the switch in language context between the second and third event (such as an English context in list 2 switching to a Spanish context in list 3) produced a reliable memory advantage for the English-Spanish bilinguals (relative to the control participants). The results offer preliminary evidence that task-irrelevant language context can influence a bilingual’s ability to remember non-linguistic information, as well as further evidence for context effects and multi-sensory effects in memory.

## Introduction

Considering that we likely hear upwards of hundreds of words per hour (Hart and Risley, 2003), much of our encoding of visual memories occurs in the context of spoken language. For most people, this spoken language context consists of one or both of their two languages, as bilingualism is the norm worldwide (Grosjean, 2015). Despite the prevalence and despite considerable research on bilingual memory (e.g., Marian and Neisser, 2000; Pu and Tse, 2014; Basnight-Brown and Altarriba, 2016; Heredia and Cieślicka, 2019), there appears to be no published research examining whether a bilingual’s language context influences visual memory (or, more broadly, memory for non-linguistic information). The current study provides an initial examination, by assessing whether a change in language context during or between events influences English-Spanish bilinguals’ ability to remember novel shapes.

**Figure 1:**
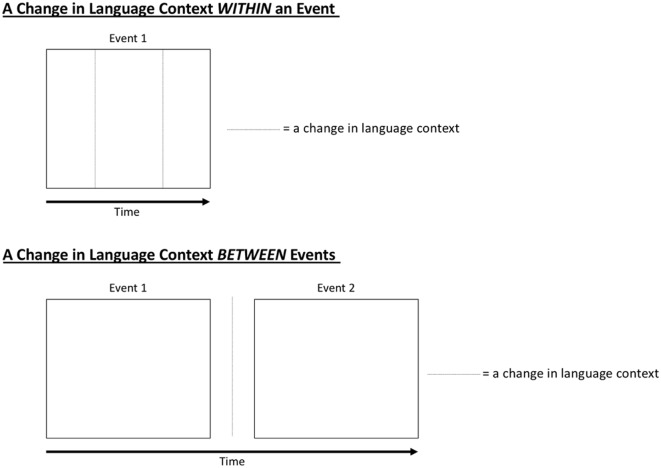
How a change in language context could affect event segmentation. This image illustrates scenarios in which a change in language context could facilitate the parsing of an experience into events and subevents. The top panel (“A Change in Language Context WITHIN an Event”) depicts a situation in which there are two changes in language context during an event (e.g., from English to Spanish and then from Spanish to English), thereby creating three subevents (i.e., a beginning, middle, and end) within the larger event. The bottom panel (“A Change in Language Context BETWEEN Events”) depicts a situation in which there is a change in language context in between two larger events, such that one language context was present during Event 1 and a different language context was present during Event 2 (e.g., English in Event 1 and Spanish in Event 2).

As a framework to guide us, we can draw upon research from the event processing literature (for reviews, see Zacks and Swallow, 2007; Kurby and Zacks, 2008; Radvansky and Zacks, 2017). According to event processing work (such the Event Horizon Model and Event Segmentation Theory), people parse their ongoing experiences into events and subevents. In other words, people segment continuous activity (e.g., watching a movie or going to the grocery store) into parts and subparts. Possibly, event segmentation could be assisted by a change in language context (for example, a switch from English into Spanish). Specifically, a change in language context *within* an event could facilitate segmentation into subevents (i.e., ending one subevent and starting a new subevent), and a change in language context *between* events could facilitate segmentation into separate events (i.e., ending one event and starting a new event; see Figure 1 below for a depiction of these two possibilities). As event segmentation is highly correlated with later event memory (Kurby and Zacks, 2008; Swallow et al., 2009; Sargent et al., 2013), changes in language context over the course of events could affect (even improve) event memory. These two possibilities—i.e., within-event and between-event changes in language context affecting visual event memory—are now fleshed out, with the within-event change discussed first and the between-event change discussed second.

How could a change in language context *within* an event boost visual memory? In other words, why might visual memory be enhanced when there is a switch in language context at one or more points during the course of a visual event? Theories of event processing, such as the Event Horizon Model, posit that more subevents (each with fewer elements) will lead to better memory than fewer subevents (each with more elements; Radvansky, 2012; Pettijohn et al., 2016). Consistent with this hypothesis, recent work found that memory (e.g., remembering word lists) was improved when the encoding event contained a segmenting cue (e.g., walking through a doorway from one room to another, or closing and then opening a computer window), which divided the encoding event into subevents (Pettijohn et al., 2016). Furthermore, more segmenting cues (and thus more subevents) led to better memory. The explanation is that segmenting an event into subevents provides an organizational structure and breaks the to-be-remembered information into smaller (and thus easier to remember) chunks. Following from this research, the first hypothesis assessed in the current study is that changes in language context *within* a visual event (thereby creating subevents) will enhance a bilingual’s visual memory.

It is also possible that a change in language context *between* visual events could boost visual memory (the second hypothesis). This hypothesis is rooted in the well-established finding that, when multiple similar events occur over time, there can be proactive interference from the first event to subsequent events, leading to worse memory for subsequent events (than would otherwise be observed; Postman et al., 1968; Anderson and Neely, 1996; Kane and Engle, 2000). In other words, if the first event and second event are similar, memory for the second event can be hindered. However, evidence suggests that proactive interference can be reduced if there is a change in context between the first event and the second event (or subsequent events; Sahakyan and Kelley, 2002; Pastötter and Bäuml, 2007; Bäuml and Kliegl, 2013). With the first and second events having distinct contexts, presumably the two events become easier to differentiate, thereby reducing competitive interference. Extending this line of thinking to language contexts in bilinguals, if the language context (e.g., English) in the first event differs from the language context (e.g., Spanish) in the second event, then proactive interference from the first event to the second event may be diminished. There is some evidence consistent with this hypothesis, as studies assessing word-list memory in bilinguals have shown that a language switch between lists (e.g., list 1 consisting of English words and list 2 consisting of Spanish) can reduce proactive interference and improve memory for list 2 (Goggin and Wickens, 1971; Dillon et al., 1973; Francis, 1999). The current study extends this work on verbal memory to visual memory, by examining whether a change in language context between events can enhance a bilingual’s visual memory (the second hypothesis of the study).

As an initial exploration of potential language context effects on visual memory in bilinguals, the current study asked English-Spanish bilinguals and controls (i.e., participants who did not know both English and Spanish) to encode three lists of novel shapes, with a free recall drawing test following each list. During the visual encoding of the shapes, a language context was present (i.e., each new shape was introduced with the phrase “this drawing looks like this” in either English or Spanish). The language context was entirely in English for one list of shapes (i.e., the English-Only Context), entirely in Spanish for a second list (i.e., the Spanish-Only Context), and partly in English and partly in Spanish for a third list (i.e., the English-Spanish Context), with the order of these lists counterbalanced across participants.

The first hypothesis—i.e., that a change in language context *within* an event might boost a bilingual’s visual memory—predicted better recall performance for the English-Spanish bilinguals in the English-Spanish Context (as this context involved a within-event language change from English to Spanish to English) relative to the English-Only and Spanish-Only Contexts (as these contexts did not involve a within-event language change) and relative to the control participants (who had a less-comprehensible change in language context). The second hypothesis—i.e., that a change in language context *between* visual events might boost a bilingual’s visual memory—predicted reduced proactive interference from the first list of shapes to the subsequent lists of shapes (i.e., lists 2 and 3) for the English-Spanish bilinguals, because the paradigm entailed a change in language context between each list (i.e., from list 1 to list 2 to list 3). This hypothesis might thus result in the English-Spanish bilinguals having a non-significant decline in recall performance from list 1 to subsequent lists (lists 2 and 3), as well as better performance on lists 2 and 3 relative to control participants (who had a less-comprehensible change in language context). The control participants may also benefit from a change in language context within and between events, despite not knowing one of the languages, though this benefit may be smaller.

## Materials and Methods

### Participants

Seventy young adults (mean age = 20.41 years; gender = 41 female, 29 male) were included. Participants consented to participate in the experimental protocol, and the protocol was approved by the ethics board at Hofstra University. Participants were categorized as English-Spanish Bilinguals (*N* = 38) or Controls (*N* = 32) based on a post-experiment questionnaire completed by participants. If participants listed both English and Spanish as languages they have knowledge of, then they were placed into the English-Spanish Bilingual group; if they did not list both English and Spanish, then they were placed into the Control group (this atypical group assignment process, in which participants were *post hoc* assigned to groups rather than in the recruitment phase, was chosen because it reduced the likelihood that participants and experimenters would guess their group and the purpose of the experiment).

Demographic information on the English-Spanish Bilinguals and Controls is provided in Table 1. Specifically, Table 1 provides descriptive statistics for Age, Gender, English Proficiency (self-rated receptive proficiency in English on a 0-low to 10-perfect scale), English AoA (age at which participant first started learning English), English Use (% of time participant currently uses English when they are using a spoken language), Spanish Proficiency (self-rated receptive proficiency in Spanish on a 0-low to 10-perfect scale), Spanish AoA (age at which participant first started learning Spanish), Spanish Use (% of time participant currently uses Spanish when they are using a spoken language), and L2 Proficiency (self-rated receptive proficiency on a 0-low to 10-perfect scale in their second most proficient language). Note that L2 Proficiency was set to 0 in participants who did not list a second language.

**Table 1:** Participant demographic information.

	English-Spanish Bilinguals (*N* = 38) Mean (SD)	Controls (*N* = 32) Mean (SD)	Comparison
Age	20.45 (1.25)	20.38 (1.43)	*t*_(68)_ = 0.22, *p* = 0.82
Gender	25 Female,	16 Female,	${\text{X}}_{\left(1\right)}^{2}$ = 1.79, *p* = 0.18
	13 Male	16 Male
English proficiency	9.50 (0.69)	9.58 (0.72)*	*t*_(67)_ = 0.47, *p* = 0.64
English AoA	1.55 (2.39)	2.11 (3.78)*	*t*_(67)_ = 0.75, *p* = 0.46
English use	87.29 (15.98)	87.74 (18.27)*	*t*_(67)_ = 0.11, *p* = 0.91
Spanish proficiency	4.57 (2.75)	—	—
Spanish AoA	10.74 (4.93)	—	—
Spanish use	5.74 (9.53)	—	—
L2 proficiency	6.16 (2.72)	5.40 (3.38)*	*t*_(67)_ = 1.03, *p* = 0.31

*Note that Language Proficiency, AoA, and Use data are missing for one Control participant.

For the 38 English-Spanish Bilinguals, English was the L1 for 33 of the participants, the L2 for 4, and the L3+ for 1, whereas Spanish was the L1 for 2 of the participants, the L2 for 19, and the L3+ for 17. Note that L1, L2, and L3+ designations were determined by proficiency. In the case of a tie in proficiency, AoA was used to break the tie; if the tie remained, English was given priority because of the English-dominant context in which the participants resided. Of the 38 English-Spanish Bilinguals, 13 only listed English and Spanish as languages they know, whereas 25 listed more than English and Spanish.

Among the 32 Controls, five only listed English, whereas 27 listed more languages than English. English was the L1 for 28 of the Controls, the L2 for 3, and the L3+ for 1. The Controls listed a wide variety of languages, including Romance languages (i.e., a language that derived from Latin, such as French, Portuguese, Italian, Romanian, and Catalan). Nineteen of the Controls listed knowledge of a Romance language. Possibly, knowledge of a Romance language could lead to partial understanding of the Spanish that was heard in the experiment, thereby affecting recall performance. However, the Romance Controls and Non-Romance Controls patterned similarly on the key finding reported in the Results section below. That is, both groups showed a clear proactive interference pattern (recall percentage for Romance Controls: List 1 = 53%, List 2 = 48%, List 3 = 41%; recall percentage for Non-Romance Controls: List 1 = 49%, List 2 = 45%, List 3 = 42%).

Note that the use of the term “bilingualism” in the current study is based on the inclusive and minimalist definition by Mackey (1962): a bilingual is a person who has “the ability to use more than one language.” However, given that bilingualism is a label with “open-ended semantics” (Baetens Beardsmore, 1982) and many definitions, some readers of the current study might have a stricter definition of bilingualism; in such a case, “second language learners” would be a more suitable label for the English-Spanish speakers.

### Procedure

The memory task involved encoding three lists of novel shapes, with a drawing-based free recall test following each list. Each of the three lists had a different language context: (1) English-Only Context; (2) Spanish-Only Context; and (3) English-Spanish Context. The order of the three language contexts was counterbalanced across participants. Participants were told not to be concerned with the language context (rather, they should be concerned with memorizing the novel shapes). The specifics of encoding and retrieval are provided below.

#### Encoding

In each of the three encoding lists, participants were presented with 12 drawings of novel shapes. Three sets of 12 drawings were created for the experiment, and each set appeared with each of the three lists (List 1, List 2, List 3) and with each of the three language contexts (English-Only Context, Spanish-Only Context, English-Spanish Context) a similar number of times.

The language context was created by introducing each novel shape with the phrase: “This drawing looks like this” (the phrase in Spanish is “Este dibujo se ve así.”). Both the English and Spanish phrase were recorded in the same voice, by an English-Spanish bilingual speaker. In the English-Only Context, the English phrase was used to introduce each (and every) shape, and likewise, in the Spanish-Only Context, the Spanish phrase was used to introduce each (and every) shape. In the English-Spanish Context, the English phrase was used to introduce shapes 1 through 4, the Spanish phrase was used to introduce shapes 5 through 8, and the English phrase was used to introduce shapes 9 through 12. There were thus two language switches rather than one, because previous work indicated that two segmenting cues (and thus three subevents) led to better memory than one segmenting cue (and thus two subevents; Pettijohn et al., 2016).

**Figure 2:**
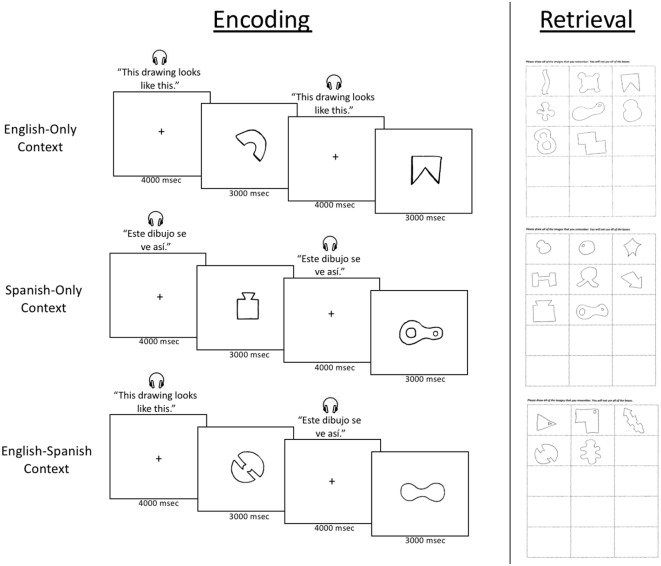
The encoding and retrieval components of the memory test. The left side depicts the encoding procedure, in which participants viewed a series of novel shapes (in an English-Only, Spanish-Only, or English-Spanish language context). The right side depicts the retrieval procedure, in which participants drew all of the novel shapes they remembered seeing in the preceding encoding procedure. Note that the order of the three language contexts was counterbalanced across participants (as were the sets of shapes).

The phrase “This drawing looks like this” was heard during the 4,000 ms interstimulus interval before each shape appeared. After the 4,000 ms interstimulus interval, the shape appeared in the center of the screen for 3,000 ms. Figure 2, on the left side, provides a visual depiction of the encoding process.

Before each encoding list, participants were instructed to learn the novel shapes for a memory test but not to be concerned with the language context. Specifically, the instructions were: “You are going to see a string of abstract images. Try to remember as many images as you can; you will be asked to draw them after the recording ends. You will also hear someone talking in English or Spanish; you can ignore the person talking, as you will not have to remember what they said or what language they were using.”

#### Retrieval

Immediately after each encoding list, participants were asked to perform a free recall test in which they were to draw all of the shapes they remembered from the preceding list of 12 shapes. The recall sheet that was used can be seen on the right side of Figure 2. The recall sheet reads: “Please draw all of the images that you remember. You will not use all of the boxes.” Participants were given an unlimited amount of time to complete the free recall test.

After completing the memory task, participants filled out the post-experiment language and demographic questionnaire. The memory task data were coded by a research assistant who was blind to the participant’s group and to the purpose of the experiment. The coding entailed matching each of the participant’s drawings to one of the drawings in the target list. If the drawing could be exclusively matched to a single drawing in the target list (even if some minor details were missing), the participants received credit for that drawing; however, if the drawing could not be matched to any drawings, could be matched to multiple drawings, or could be matched to a drawing in a non-target list, the participant did not receive credit.

Consistent with the values of open science, the raw visual memory data, the recall scores, and the relevant questionnaire data are freely available to the public through the Open Science Framework at the following web address: https://osf.io/f5wrh/. All other information and stimuli will be willingly provided by the author.

## Results

### A Change in Language Context Within an Event

**Figure 3:**
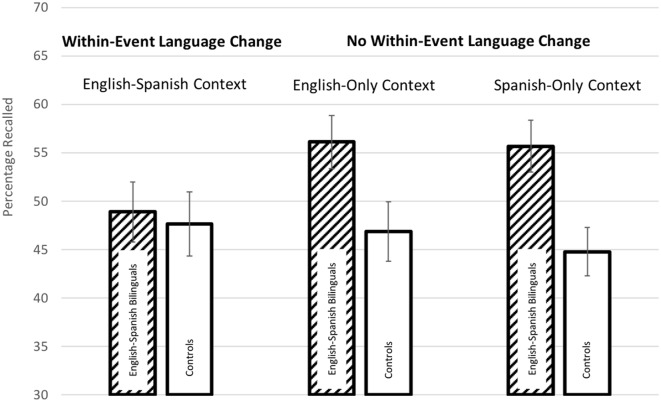
Visual memory performance when there was (and was not) a change in language context within an event. This image displays visual memory performance (percentage of shapes recalled) for English-Spanish Bilinguals and Controls when there was a change in language context within an event (English-Spanish Context) and when there was no change in language context within an event (English-Only Context and Spanish-Only Context).

To assess the *first hypothesis* (i.e., that a change in language context *within* an event might help visual memory in bilinguals), we can compare recall when there was a change in language context within an event (i.e., the English-Spanish Context) to recall when there was not (i.e., the English-Only Context and the Spanish-Only Context) in the English-Spanish Bilinguals and the Controls. These data are displayed in Figure 3 below. As can be seen in Figure 3, the mean percentage of shapes recalled for the English-Spanish Bilinguals was *not* higher when there was a change in language context within an event (i.e., the English-Spanish Context) vs. when there was not; in fact, the opposite pattern was seen. Furthermore, English-Spanish Bilinguals’ recall when there was a within-event language context change (i.e., the English-Spanish Context) appears to be very similar to that of Controls’ recall. Thus, by visual inspection, the data clearly do not support the hypothesis that a change in language context within an event helps visual memory in bilinguals.

Two statistical tests were conducted to assess the first hypothesis: a traditional ANOVA and a generalized linear mixed-effects model.

#### ANOVA

The ANOVA was a 3 × 2, with Language Context (English-Spanish Context vs. English-Only Context vs. Spanish-Only Context) as a within-subjects independent variable, Group (English-Spanish Bilinguals vs. Controls) as a between-subjects independent variable, and mean percentage of shapes recalled as the dependent variable. The ANOVA yielded a non-significant main effect of Language Context, *F*_(2,136)_ = 1.06, *p* = 0.35, partial *η*^2^ = 0.02, a significant main effect of Group, *F*_(1,68)_ = 4.59, *p* = 0.04, partial *η*^2^ = 0.06, and a non-significant interaction between Language Context and Group, *F*_(2,136)_ = 2.13, *p* = 0.12, partial *η*^2^ = 0.03. The significant main effect of Group reflects an advantage for the English-Spanish Bilinguals, but this advantage seems to be due mostly to enhanced performance when there was *no* change in language context within an event, which goes *against* the hypothesis.

#### Mixed-Effects Model

A generalized linear mixed-effects model yielded similar results. The model consisted of Group and Language Context as fixed effects and Participant as a random effect (on the intercept). The model was computed using the glmer function in R, with the fixed effects sum coded, and with significance assessed through an Analysis of Deviance Table (Type III Wald chi-square tests). There was a non-significant main effect of Language Context, $\chi_{\left(2\right)}^{2}$ = 1.73, *p* = 0.42, a significant main effect of Group, $\chi_{\left(1\right)}^{2}$ = 4.62, *p* = 0.03, and a trending interaction between Language Context and Group, $\chi_{\left(2\right)}^{2}$ = 5.39, *p* = 0.07.

### A Change in Language Context Between Events

**Figure 4:**
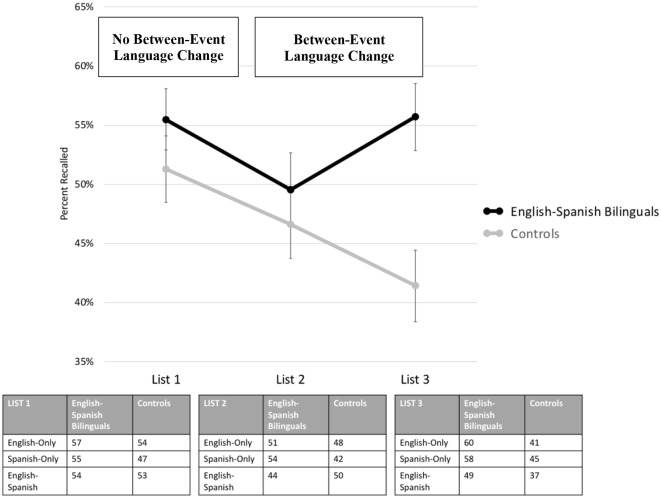
Visual memory performance when there was (and was not) a change in language context between events. This image displays visual memory performance for English-Spanish Bilinguals (top line) and Controls (bottom line) when there was no between-event language context change (i.e., List 1) and when there was a between-event language context change (i.e., Lists 2 and 3). The mean percentage of shapes recalled for the three language contexts (English-Only, Spanish-Only, and English-Spanish) are displayed at the bottom.

To assess the *second hypothesis* (i.e., that a change in language context *between* events might help visual memory in bilinguals), we can compare recall when there was a change in language context between events (i.e., Lists 2 and 3) to recall when there was not (i.e., List 1) in the English-Spanish Bilinguals and the Controls. These data are shown in Figure 4 below. A visual inspection of Figure 4 reveals a consistent decline in recall for the Controls from List 1 to List 2 to List 3, i.e., a proactive interference effect. For the English-Spanish Bilinguals, however, the decline is less consistent, with List 3 showing the opposite of proactive interference and resulting in a noticeable difference between the English-Spanish Bilinguals and Controls. These visual impressions are partially consistent with the second hypothesis.

As with the first hypothesis, two statistical tests (i.e., a traditional ANOVA and generalized linear mixed-effects model) were used to assess the second hypothesis.

#### ANOVA

The 3 × 2 ANOVA had List Number (List 1 vs. List 2 vs. List 3) as a within-subjects independent variable, Group (English-Spanish Bilinguals vs. Controls) as a between-subjects independent variable, and mean percentage of shapes recalled as the dependent variable. The ANOVA revealed a significant main effect of List Number, *F*_(2,136)_ = 3.37, *p* = 0.04, partial *η*^2^ = 0.05, reflecting a proactive interference effect from List 1 to List 2 to List 3, and a significant main effect of Group, *F*_(1,68)_ = 5.13, *p* = 0.03, partial *η*^2^ = 0.07, reflecting that English-Spanish Bilinguals had better recall overall than Controls. Crucially, there was also a significant interaction between List Number and Group, *F*_(2,136)_ = 3.79, *p* = 0.03, partial *η*^2^ = 0.05.

To follow up the interaction, Bonferroni-corrected *t*-test comparisons among lists (i.e., List 1 vs. List 2, List 1 vs. List 3, and List 2 vs. List 3) were conducted for both English-Spanish Bilinguals and Controls. The only comparison that survived correction for multiple comparisons was List 1 vs. List 3 in Controls (*p* = 0.01), reflecting a significant decline in performance from List 1 to List 3 (i.e., a proactive interference effect) for Controls (but not for English-Spanish Bilinguals). The decline for the Controls in List 3, in conjunction with a reversal pattern for English-Spanish Bilinguals, appeared to create a sizable difference between groups in List 3 (but not Lists 1 and 2). To assess statistical significance, Bonferroni-corrected *t*-tests compared groups on each of the 3 lists, with the only significant difference emerging on List 3 (*p* = 0.003).

#### Mixed-Effects Model

Next, a generalized linear mixed-effects model was conducted, with Group and List Number as fixed effects and Participant as a random effect (on the intercept). The generalized linear mixed-effects model allows us to determine if the crucial interaction between Group and List Number could be replicated with a different type of analysis and with an analysis that accounts for the random effect of participants. The model was computed using the glmer function in R. The fixed effects were sum coded, and statistical significance was determined through an Analysis of Deviance Table (Type III Wald chi-square tests). The analysis yielded a trending main effect of List Number, $\chi_{\left(2\right)}^{2}$ = 5.01, *p* = 0.08, a significant main effect of Group, $\chi_{\left(1\right)}^{2}$ = 4.60, *p* = 0.03, and, critically, a significant interaction between List Number and Group, $\chi_{\left(2\right)}^{2}$ = 6.54, *p* = 0.04.

### Additional Analyses

Analyses were then conducted in order to rule out alternative explanations for the finding of superior recall for English-Spanish Bilinguals relative to Controls on List 3 (resulting from a lack of proactive interference). It seemed possible that the high recall was due, not to the English-Spanish bilingualism *per se*, but either to: (1) bilingualism more generally; or (2) by chance to the order in which groups completed the lists (given the atypical group assignment process). This first alternative explanation was excluded as a likely possibility because English-Spanish Bilinguals and Controls did not differ in their second language proficiency, *t*_(67)_ = 1.03, *p* = 0.31, and second language proficiency did not correlate with recall on List 3 (*r* = 0.03, *p* = 0.81). The second alternative explanation was also excluded as a likely possibility, as a log-linear analysis of a 3-way contingency table of Group (English-Spanish Bilinguals vs. Controls), Language Context (English-Only Context vs. Spanish-Only Context vs. English-Spanish Context), and List Number (List 1 vs. List 2 vs. List 3) revealed no significant or near-significant interaction between Group, Language Context, and List Order, *G*^2^ = 2.04, *df* = 12, *p* = 0.99 (this contingency table is represented in Table 2 below). In other words, despite the atypical group assignment process, the two groups were exposed to the language contexts in a similar order.

**Table 2:** Order of conditions by group.

English-Spanish bilinguals (*N* = 38)	List 1	List 2	List 3
English-only context	14	12	12
Spanish-only context	13	13	12
English-Spanish context	11	13	14
**Controls (*N* = 32)**	**List 1**	**List 2**	**List 3**
English-only context	8	11	12
Spanish-only context	11	10	11
English-Spanish context	12	11	9

*Note. Each cell contains the number of participants who were exposed to a given language context in a given list order. For example, 14 of the 38 English-Spanish Bilinguals completed the English-Only Context in the first list*.

In a final, exploratory analysis, a potential effect of the initial language context (i.e., English vs. Spanish) on subsequent memory performance was assessed. That is, did starting in English or in Spanish on List 1 affect subsequent recall for the English-Spanish Bilinguals? To assess this question, English-Spanish Bilinguals who started on the English-Only Context (i.e., English-starters; *N* = 14) and English-Spanish Bilinguals who started on the Spanish-Only Context (i.e., Spanish-starters; *N* = 13) were compared in their performance on the subsequent single-language context (i.e., Spanish for the English-starters and English for Spanish-starters) and the English-Spanish Context (Eleven started on the English-Spanish Context and were thus not included in this analysis). The English-starters had a mean recall percentage of 53.60% (SD = 12.87%) on the single-language context and 41.07% (SD = 12.85%) on the English-Spanish Context, whereas the Spanish-starters had a mean recall percentage of 53.85% (SD = 20.30%) on the single-language context and 53.21% (SD = 25.58%) on the English-Spanish Context. Thus, numerically, the Spanish-starters performed better than the English-starters on the English-Spanish context. However, the interaction between Group (English-starters vs. Spanish-starters) and Language Context (single-language context vs. English-Spanish Context) did not reach significance in either an ANOVA, *F*_(1,25)_ = 2.49, *p* = 0.13, partial *η*^2^ = 0.09, or a generalized linear mixed-effects model with Participant as a random effect (same model details as the above mixed-effects models), $\chi_{\left(1\right)}^{2}$ = 2.32, *p* = 0.13.

## Discussion

With research on event processing as a guiding theoretical framework, the current study served as a preliminary examination into how changes in the ambient linguistic environment might influence visual memory in bilinguals. Specifically, the study assessed whether a shift in language context within an event (hypothesis 1) or between events (hypothesis 2) enhances a bilingual’s visual memory, with the results providing partial initial empirical support for hypothesis 2 (but not hypothesis 1). In partial support of hypothesis 2, the control participants had a consistent downward recall trajectory from the first list to the second list to the third list (i.e., a proactive interference effect), whereas the English-Spanish bilinguals did not have a decline from the second list to the third list (resulting in a recall advantage on the third list for the English-Spanish bilinguals relative to the controls), presumably because of the change in language context between lists. Thus, while merely preliminary, the results suggest that the ambient linguistic background may in some circumstances boost a bilingual’s non-linguistic memory performance.

Although the results are consistent with hypothesis 2 (i.e., that a change in language context between events helps memory), they are only partially so, because the memory benefit emerged on the third list but not the second list. Why did the benefit emerge only on the third list? A plausible explanation is that by the onset of the third list, participants had been exposed to two *different* language contexts, thereby making it clear that the language context changes from list to list and could thus be used to differentiate lists. At the onset of the second list, with exposure to only one list-wide language context, participants did not know that language context would be varied across lists and that it could be used as a distinguishing element to reduce interference.

Notably, the memory benefit on the third list for the English-Spanish bilinguals appears to have been driven more by the single-language contexts (i.e., the English-Only and Spanish-Only Contexts) than the dual-language context (i.e., English-Spanish Context; see bottom of Figure 4). Why is this the case? It could be due to the single-language contexts being more distinct from the previous lists. The single-language contexts only share contextual commonalities with one of the previous lists, whereas the dual-language context shares contextual commonalities with both of the previous lists. With more dissimilarity, there is potentially less competition and better memory. A related explanation invokes language-dependent memory (Marian and Neisser, 2000; Marian and Fausey, 2006; Marian and Kaushanskaya, 2007), a phenomenon whereby a language context evokes memories that were encoded in that same language context. In the current paradigm, the single-language contexts would only cue memories of a subset of the previously encoded shapes, whereas a dual-language context would cue memories of all previously encoded shapes, potentially creating more interference.

While there was support for the second hypothesis, there was no support for the first hypothesis. Why did the results fail to support the first hypothesis? That is, why did a within-event language context change not increase memory performance? One possibility is that this benefit is restricted to high proficiency (and high use) bilinguals. Spanish proficiency was low (as was current Spanish use) for many of the English-Spanish bilinguals in the current study, as can be gleaned from the Spanish proficiency (and Spanish use) mean and standard deviation in Table 1. However, an exploratory correlation analysis reveals no link between Spanish proficiency and recall performance in the English-Spanish Context (i.e., the within-event context change condition) for the English-Spanish bilinguals (Pearson’s *r* = 0.02), suggesting that the low proficiency of many of the bilinguals did not prevent support for hypothesis 1 from emerging. Nevertheless, a follow-up study with high proficiency (and high use) bilinguals is warranted. A second possibility is that, while proficiency may not be especially relevant, code-switching behavior may be, and the potential memory enhancement from a within-event language switch may be restricted to bilinguals who code-switch frequently. A third possibility is that bilinguals incurred a cognitive processing cost when a within-event language switch occurred; that is, bilinguals may have deployed cognitive control resources to suppress the previous language (Philipp and Huestegge, 2015; Olson, 2017; but see Declerck et al., 2019), resulting in fewer resources available for memory encoding. A fourth possibility relates to the strength of the language context; perhaps the ambient linguistic context needs to be stronger and may even need to include expressive language in addition to receptive language. A fifth and final possibility is that there is a benefit to a within-event language switch, but that it was masked by a potential benefit of the single-language English-Only and Spanish-Only contexts. In other words, a consistent and meaningful context in the form of a single-language context may have aided encoding, which concealed a benefit that may also be derived from a language switch. As this list shows, there are many possibilities for why a within-event language context change effect did not manifest in the current paradigm, rendering this study preliminary and warranting additional studies.

Given that this study served as merely an initial foray into this research topic, there were several limitations (four of which are noted here) that should be addressed with future research. One shortcoming is the limited data on the linguistic backgrounds of the bilinguals (such as whether they code-switch often and are objectively proficient in the two languages). A second shortcoming is the absence of repeated language contexts (such as an English-Only Context followed by an English-Only Context) and a no-language context. A third shortcoming is that cognitive abilities, such as IQ and working memory, were not measured and thus may have differed between groups. A fourth shortcoming is that the retrieval task of drawing shapes was not completely language-free, as instructions were provided in English.

Despite these limitations, the current work provides initial data suggesting that a bilingual’s non-linguistic memory can be influenced (and even boosted) by a subtle and task-irrelevant linguistic context. On a practical level, these data imply a possible way to enhance memory, such as when studying for tests. Potentially, studying for a course’s first exam in one language context and for the second exam in a different language context could prove beneficial. On a theoretical level, these data provide further evidence that memory is influenced both by context (Smith and Vela, 2001) and by multi-sensory audio-visual interactions (Thelen et al., 2015). More broadly, the current data underline the tight link between two of our most cherished mental abilities—language and memory.

## Data Availability Statement

The data are available through the Open Science Framework at the following web address: https://osf.io/f5wrh/.

## Ethics Statement

The studies involving human participants were reviewed and approved by Hofstra University Institutional Review Board. The patients/participants provided their written informed consent to participate in this study.

## Author Contributions

SS conceived the study, designed the study, analyzed the data, wrote and revised the manuscript.

## Conflict of Interest

The author declares that the research was conducted in the absence of any commercial or financial relationships that could be construed as a potential conflict of interest.

## References

[B1] Anderson M. C. Neely J. H. (1996). Interference and inhibition in memory retrieval. in Memory eds E. L. Bjork and R. A. Bjork (Cambridge, MA: Academic Press), 237–313. 10.1016/b978-012102570-0/50010-0

[B2] Baetens Beardsmore H. (1982). Bilingualism: Basic Principles. Clevedon: Multilingual Matters.

[B3] Basnight-Brown D. M. Altarriba J. (2016). Multiple translations in bilingual memory: Processing differences across concrete, abstract and emotion words. J. Psycholinguist. Res. 45, 1219–1245. 10.1007/s10936-015-9400-4 26519144

[B4] Bäuml K.-H. T. Kliegl O. (2013). The critical role of retrieval processes in release from proactive interference. J. Mem. Lang. 68, 39–53. 10.1016/j.jml.2012.07.006

[B5] Declerck M. Koch I. Duñabeitia J. A. Grainger J. Stephan D. N. (2019). What absent switch costs and mixing costs during bilingual language comprehension can tell us about language control. J. Exp. Psychol. Hum. Percept. Perform. 45, 771–789. 10.1037/xhp0000627 30920253

[B6] Dillon R. F. McCormack P. D. Petrusic W. M. Cook G. M. Lafleur L. (1973). Release from proactive interference in compound and coordinate bilinguals. Bull. Psychon. Soc. 2, 293–294. 10.3758/bf03329277

[B7] Francis W. S. (1999). Cognitive integration of language and memory in bilinguals: semantic representation. Psychol. Bull. 125, 193–222. 10.1037/0033-2909.125.2.193 10087936

[B8] Goggin J. Wickens D. D. (1971). Proactive interference and language change in short-term memory. J. Verbal Learning Verbal Behav. 10, 453–458. 10.1016/S0022-5371(71)80046-4

[B9] Grosjean F. (2015). Bicultural bilinguals. Int. J. Biling. 19, 572–586. 10.1177/1367006914526297

[B10] Hart B. Risley T. R. (2003). The early catastrophe: The 30 million word gap by age 3. Am. Educ. 27, 4–9.

[B11] Heredia R. R. Cieślicka A. B. (2019). Bilingual episodic memory: encoding and storage. Tesol Encyclopedia Eng. Lang. Teach. 1–7. 10.1002/9781118784235.eelt0958

[B12] Kane M. J. Engle R. W. (2000). Working-memory capacity, proactive interference and divided attention: Limits on long-term memory retrieval. J. Exp. Psychol. Learn. Mem. Cogn. 26, 336–358. 10.1037/0278-7393.26.2.336 10764100

[B13] Kurby C. A. Zacks J. M. (2008). Segmentation in the perception and memory of events. Trends Cogn. Sci. 12, 72–79. 10.1016/j.tics.2007.11.004 18178125 PMC2263140

[B14] Mackey W. F. (1962). The description of bilingualism. Can. J. Linguist. Revue 7, 51–85. 10.1017/S0008413100019393

[B15] Marian V. Fausey C. M. (2006). Language-dependent memory in bilingual learning. Appl. Cogn. Psychol. Off. J. Soc. Appl. Res. Mem. Cogn. 20, 1025–1047. 10.1002/acp.1242

[B16] Marian V. Kaushanskaya M. (2007). Language context guides memory content. Psychon. Bull. Rev. 14, 925–933. 10.3758/bf03194123 18087961

[B17] Marian V. Neisser U. (2000). Language-dependent recall of autobiographical memories. J. Exp. Psychol. Gen. 129, 361–368. 10.1037/0096-3445.129.3.361 11006905

[B18] Olson D. J. (2017). Bilingual language switching costs in auditory comprehension. Lang. Cogn. Neurosci. 32, 494–513. 10.1080/23273798.2016.1250927

[B19] Pastötter B. Bäuml K.-H. (2007). The crucial role of postcue encoding in directed forgetting and context-dependent forgetting. J. Exp. Psychol. Learn. Mem. Cogn. 33, 977–982. 10.1037/0278-7393.33.5.977 17723074

[B20] Pettijohn K. A. Thompson A. N. Tamplin A. K. Krawietz S. A. Radvansky G. A. (2016). Event boundaries and memory improvement. Cognition 148, 136–144. 10.1016/j.cognition.2015.12.013 26780472

[B21] Philipp A. M. Huestegge L. (2015). Language switching between sentences in reading: exogenous and endogenous effects on eye movements and comprehension. Biling. Lang. Cogn. 18, 614–625. 10.1017/s1366728914000753

[B22] Postman L. Stark K. Fraser J. (1968). Temporal changes in interference. J. Verbal Learning Verbal Behav. 7, 672–694. 10.1016/S0022-5371(68)80124-0

[B23] Pu X. Tse C.-S. (2014). “The revised hierarchical model: Explicit and implicit memory,” in Foundations of Bilingual Memory (New York, NY: Springer), 147–184. 10.1007/978-1-4614-9218-4_8

[B24] Radvansky G. A. (2012). Across the event horizon. Curr. Dir. Psychol. Sci. 21, 269–272. 10.1177/0963721412451274

[B25] Radvansky G. A. Zacks J. M. (2017). Event boundaries in memory and cognition. Curr. Opin. Behav. Sci. 17, 133–140. 10.1016/j.cobeha.2017.08.006 29270446 PMC5734104

[B26] Sahakyan L. Kelley C. M. (2002). A contextual change account of the directed forgetting effect. J. Exp. Psychol. Learn. Mem. Cogn. 28, 1064–1072. 10.1037/0278-7393.28.6.1064 12450332

[B27] Sargent J. Q. Zacks J. M. Hambrick D. Z. Zacks R. T. Kurby C. A. Bailey H. R.. (2013). Event segmentation ability uniquely predicts event memory. Cognition 129, 241–255. 10.1016/j.cognition.2013.07.002 23942350 PMC3821069

[B28] Smith S. M. Vela E. (2001). Environmental context-dependent memory: a review and meta-analysis. Psychon. Bull. Rev. 8, 203–220. 10.3758/bf03196157 11495110

[B29] Swallow K. M. Zacks J. M. Abrams R. A. (2009). Event boundaries in perception affect memory encoding and updating. J. Exp. Psychol. Gen. 138, 236–257. 10.1037/a0015631 19397382 PMC2819197

[B30] Thelen A. Talsma D. Murray M. M. (2015). Single-trial multisensory memories affect later auditory and visual object discrimination. Cognition 138, 148–160. 10.1016/j.cognition.2015.02.003 25743256

[B31] Zacks J. M. Swallow K. M. (2007). Event segmentation. Curr. Dir. Psychol. Sci. 16, 80–84. 10.1111/j.1467-8721.2007.00480.x 22468032 PMC3314399

